# Supplementary figures and images for: Prenylated quinolinecarboxylic acid compound-18 prevents sensory nerve fiber outgrowth through inhibition of the interleukin-31 pathway
Source: PLoS One. 2021 Feb 4;16(2):e0246630. doi: 10.1371/journal.pone.0246630 (PMC7861556; doi:10.1371/journal.pone.0246630)

Fig. 3A

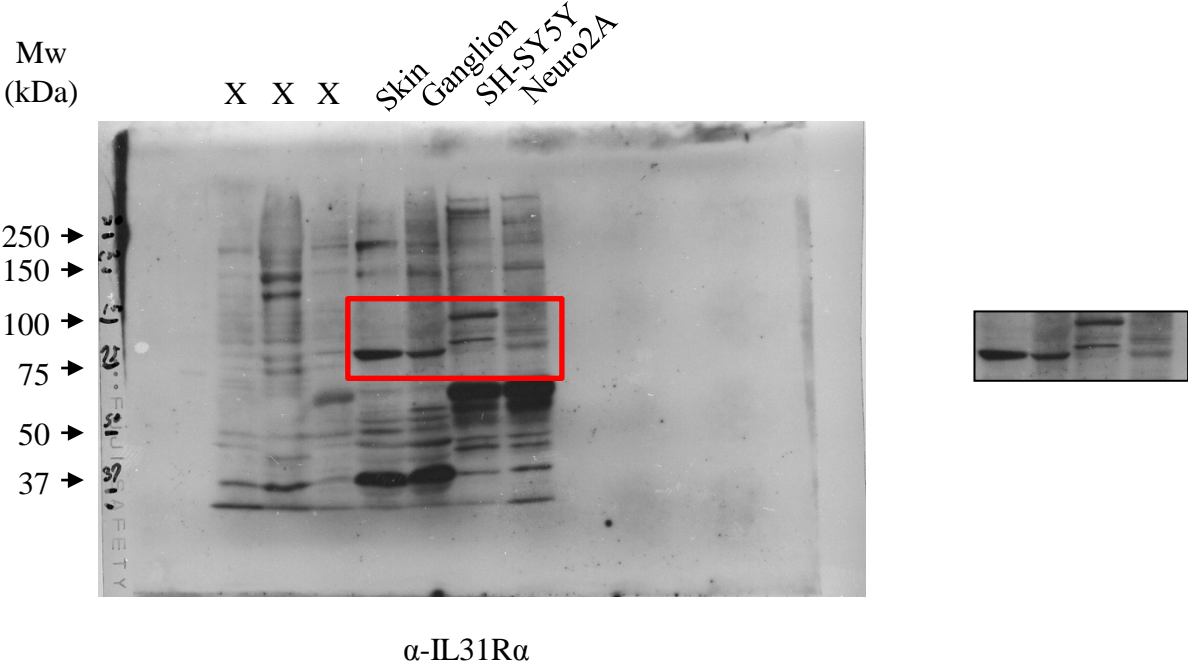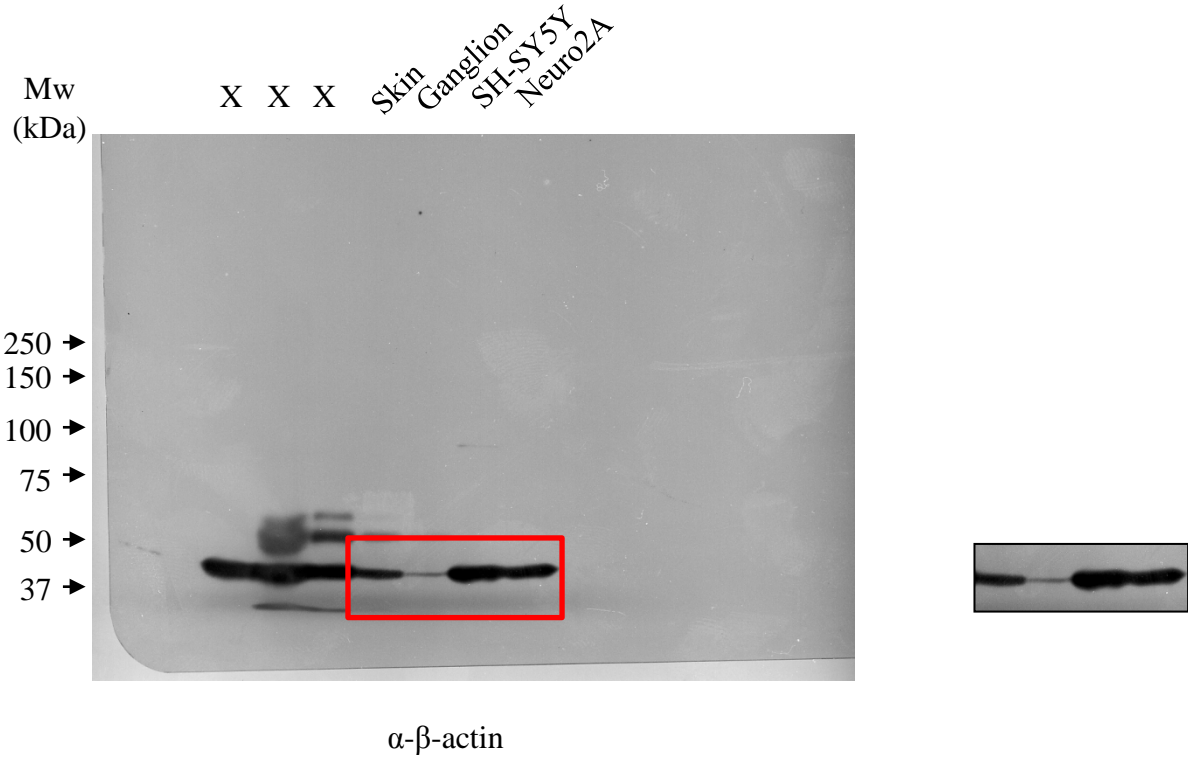

Supplement: S1 Fig — (PDF) [file pone.0246630.s001.pdf]

Fig. 3C

Mw  
(kDa)

rIL31 (ng/ml)

X X X X 0 0.1 1 10 100

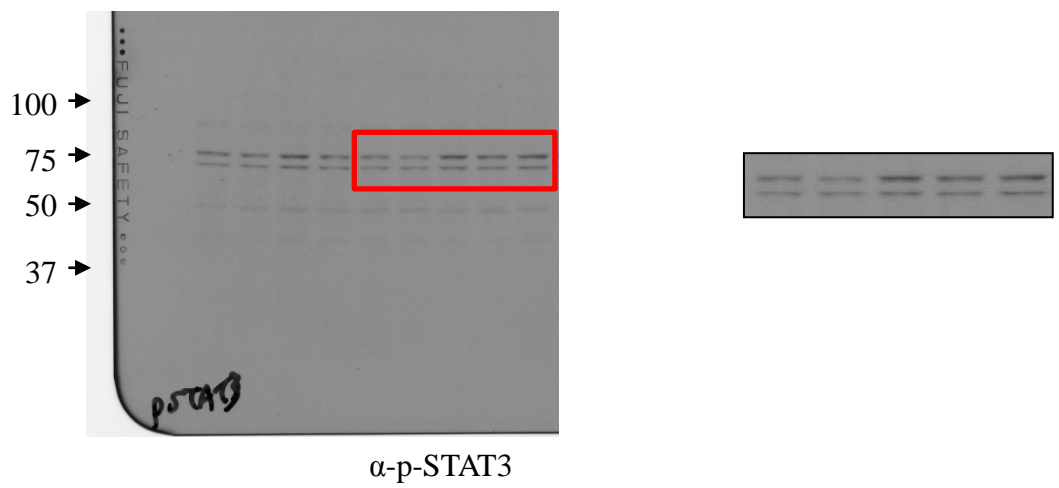

Mw  
(kDa)

rIL31 (ng/ml)

X X X X 0 0.1 1 10 100

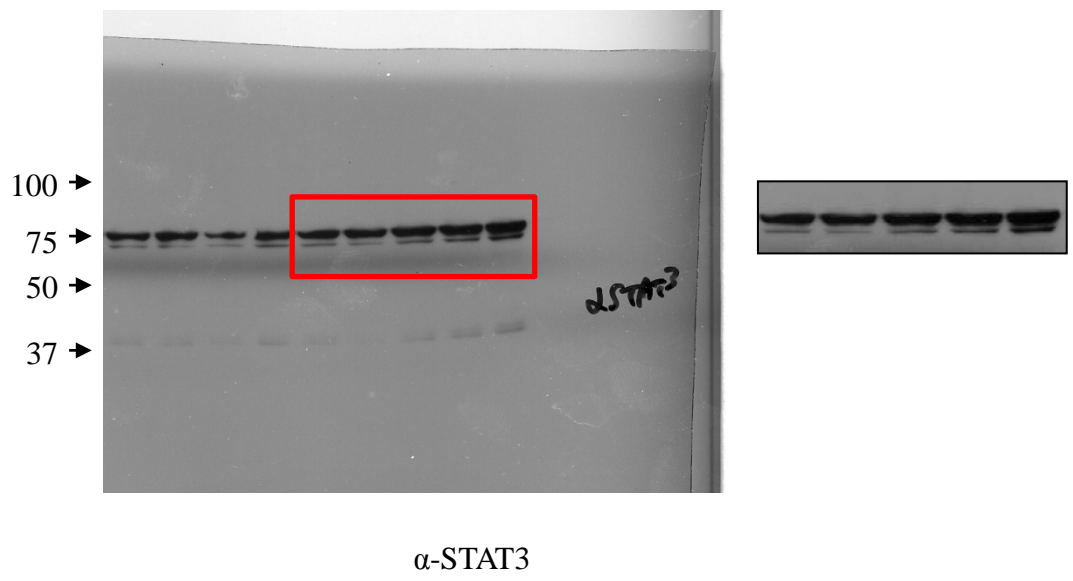

Supplement: S2 Fig — (PDF) [file pone.0246630.s002.pdf]

Fig. 3D

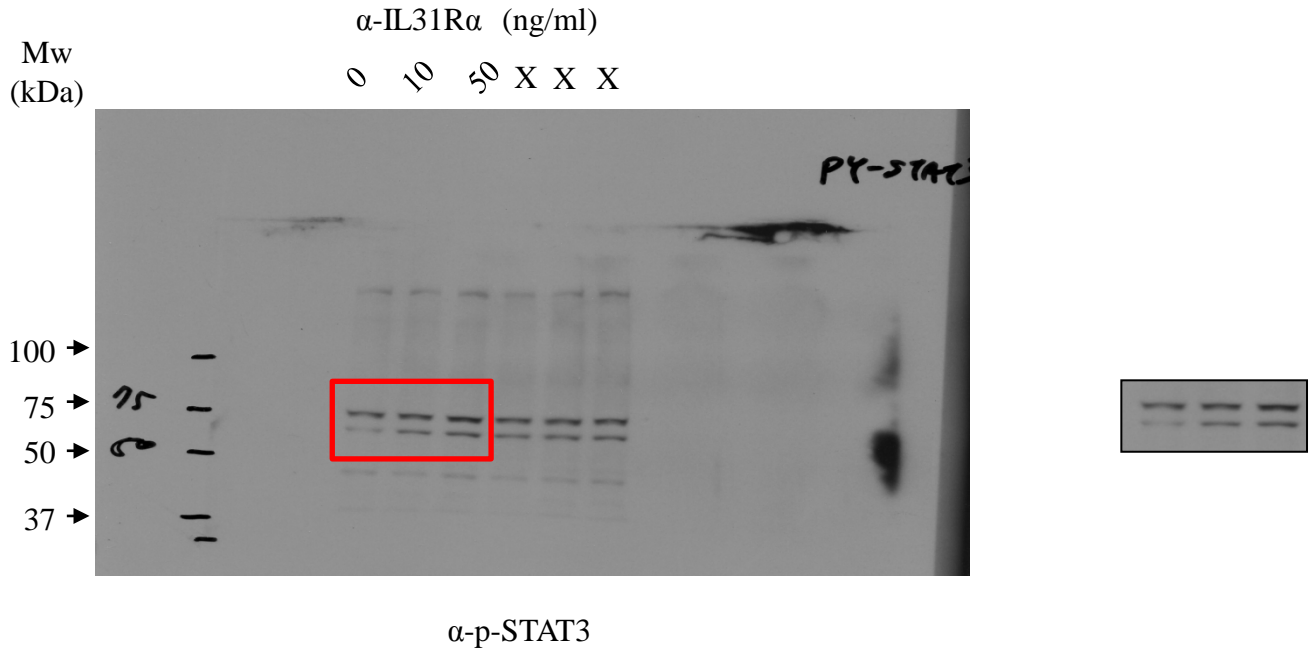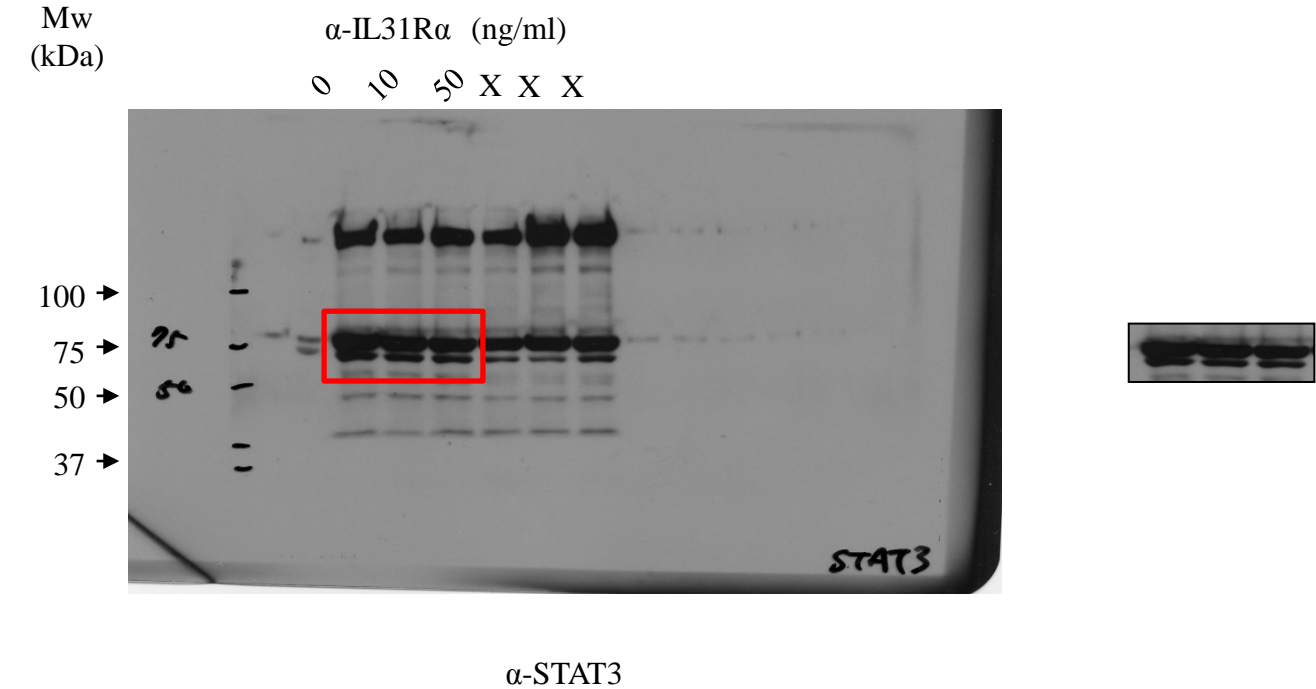

Supplement: S3 Fig — (PDF) [file pone.0246630.s003.pdf]

Fig. 4a

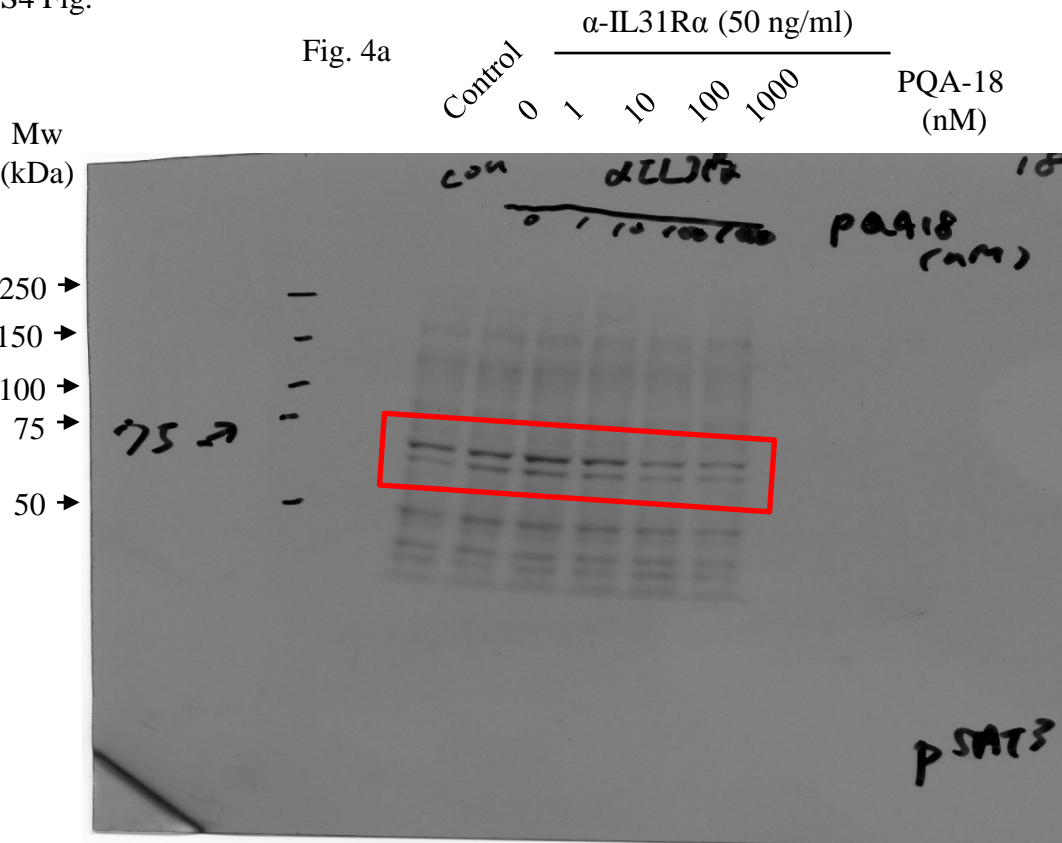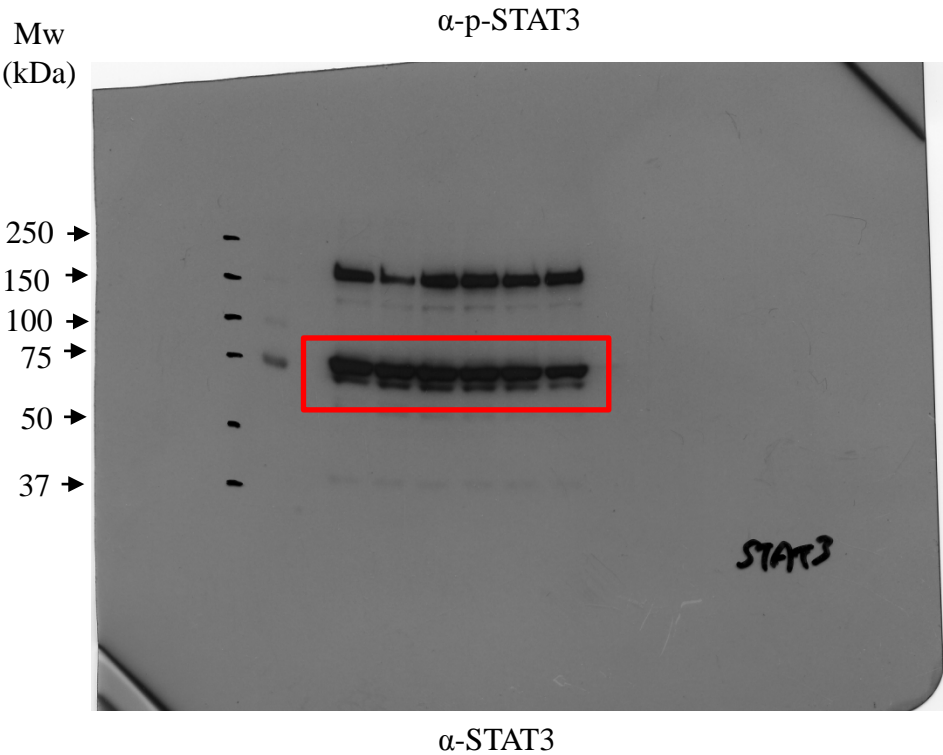

Supplement: S4 Fig — (PDF) [file pone.0246630.s004.pdf]

Fig. 4b

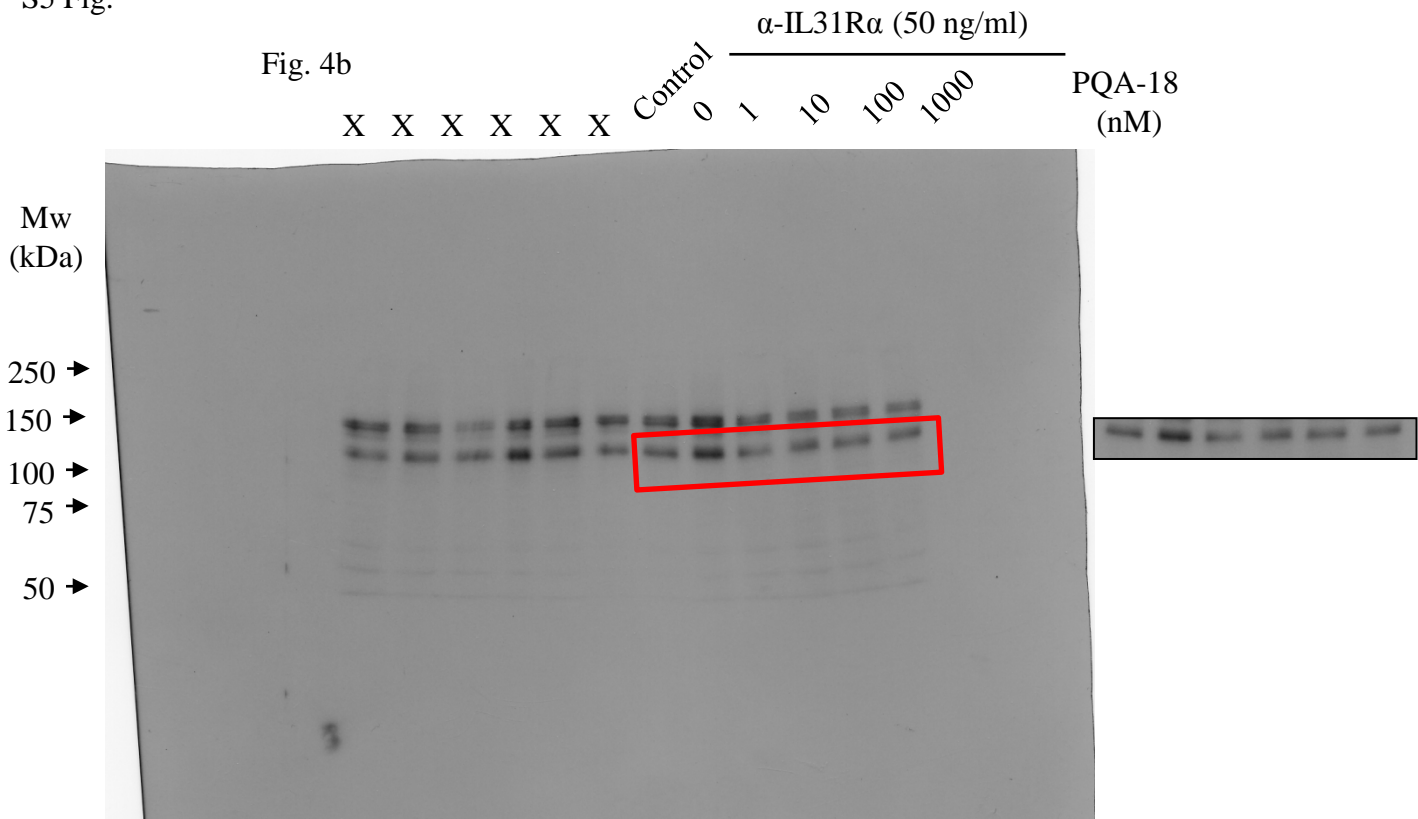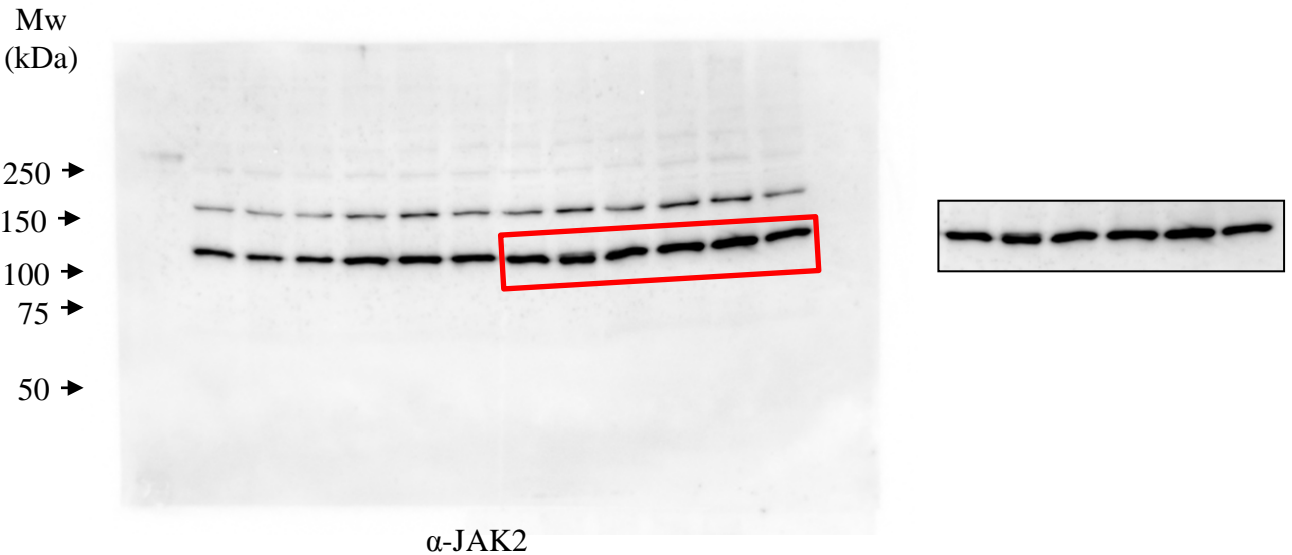

Supplement: S5 Fig — (PDF) [file pone.0246630.s005.pdf]

Fig. 4c

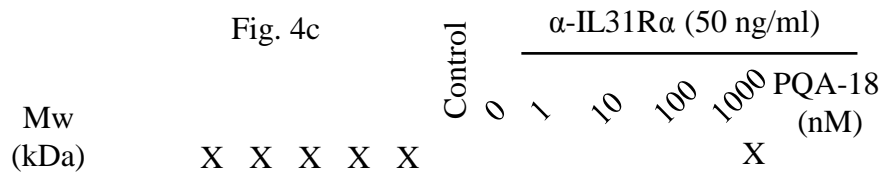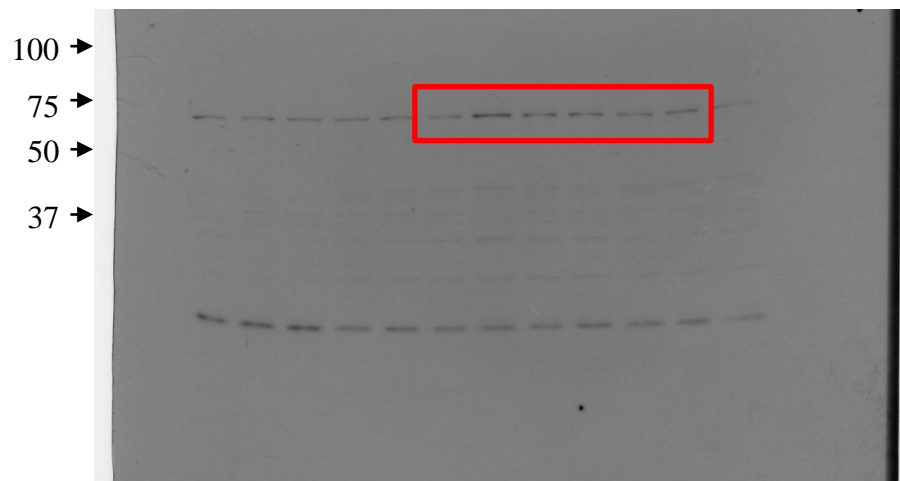 $\alpha$ -p-PAK2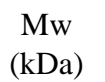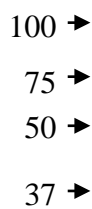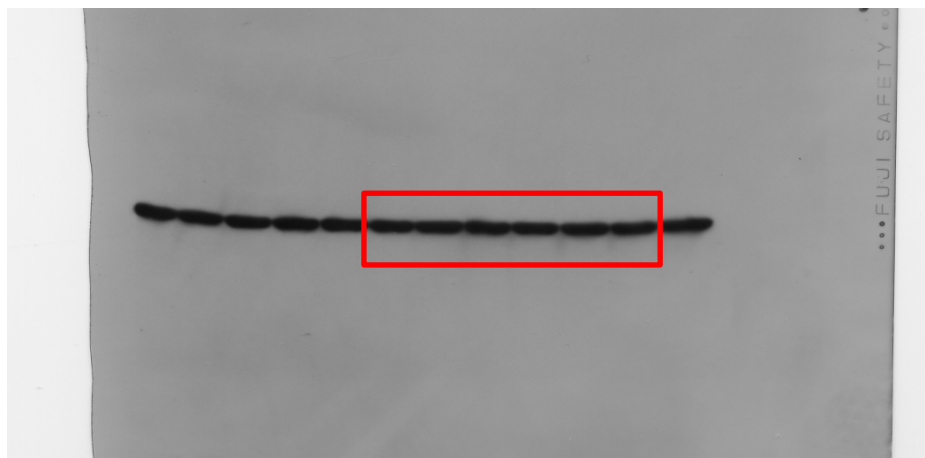

$\alpha$ -PAK2

Supplement: S6 Fig — (PDF) [file pone.0246630.s006.pdf]

Fig. 6A

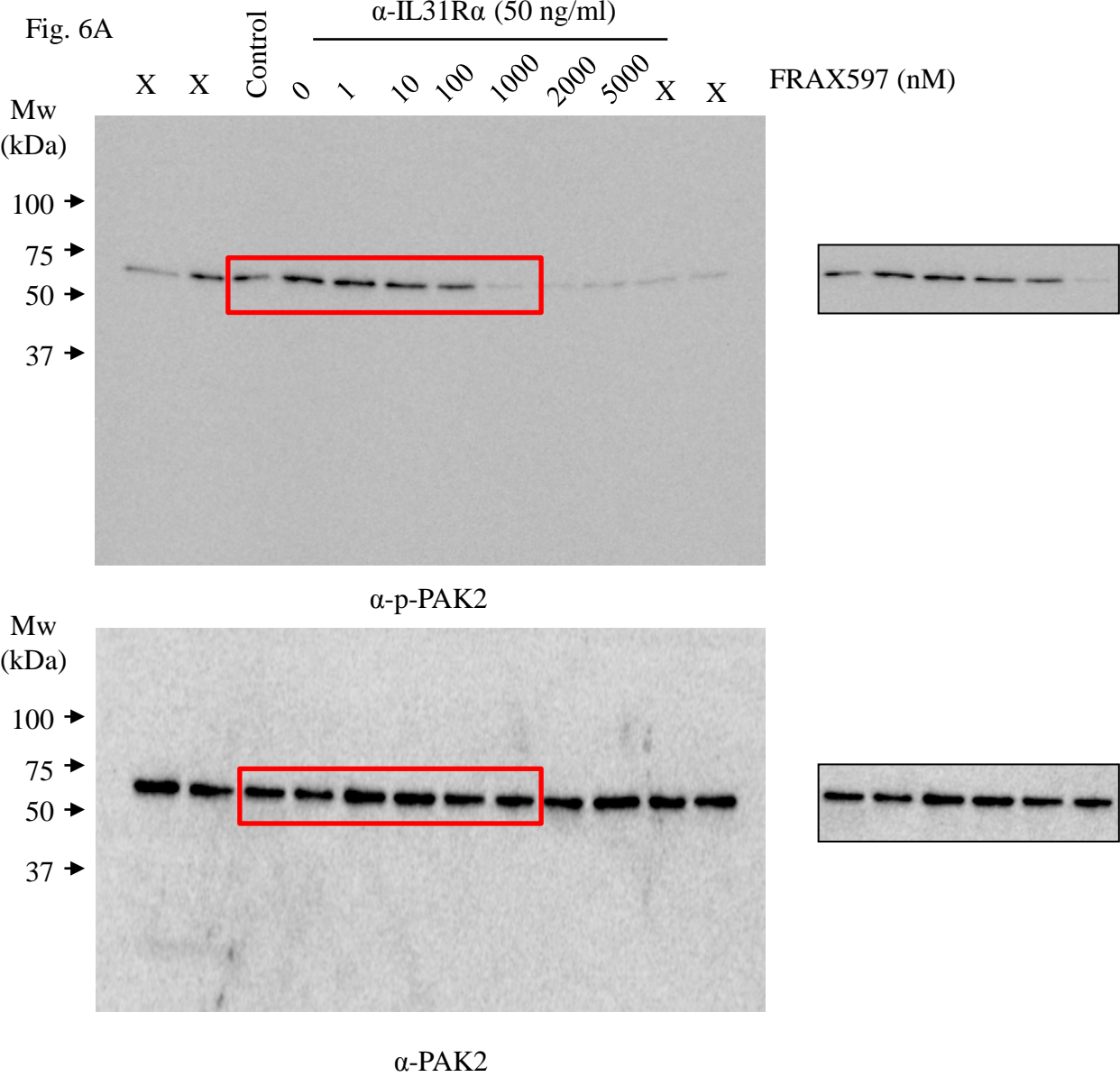

Supplement: S7 Fig — (PDF) [file pone.0246630.s007.pdf]

Fig. 7A

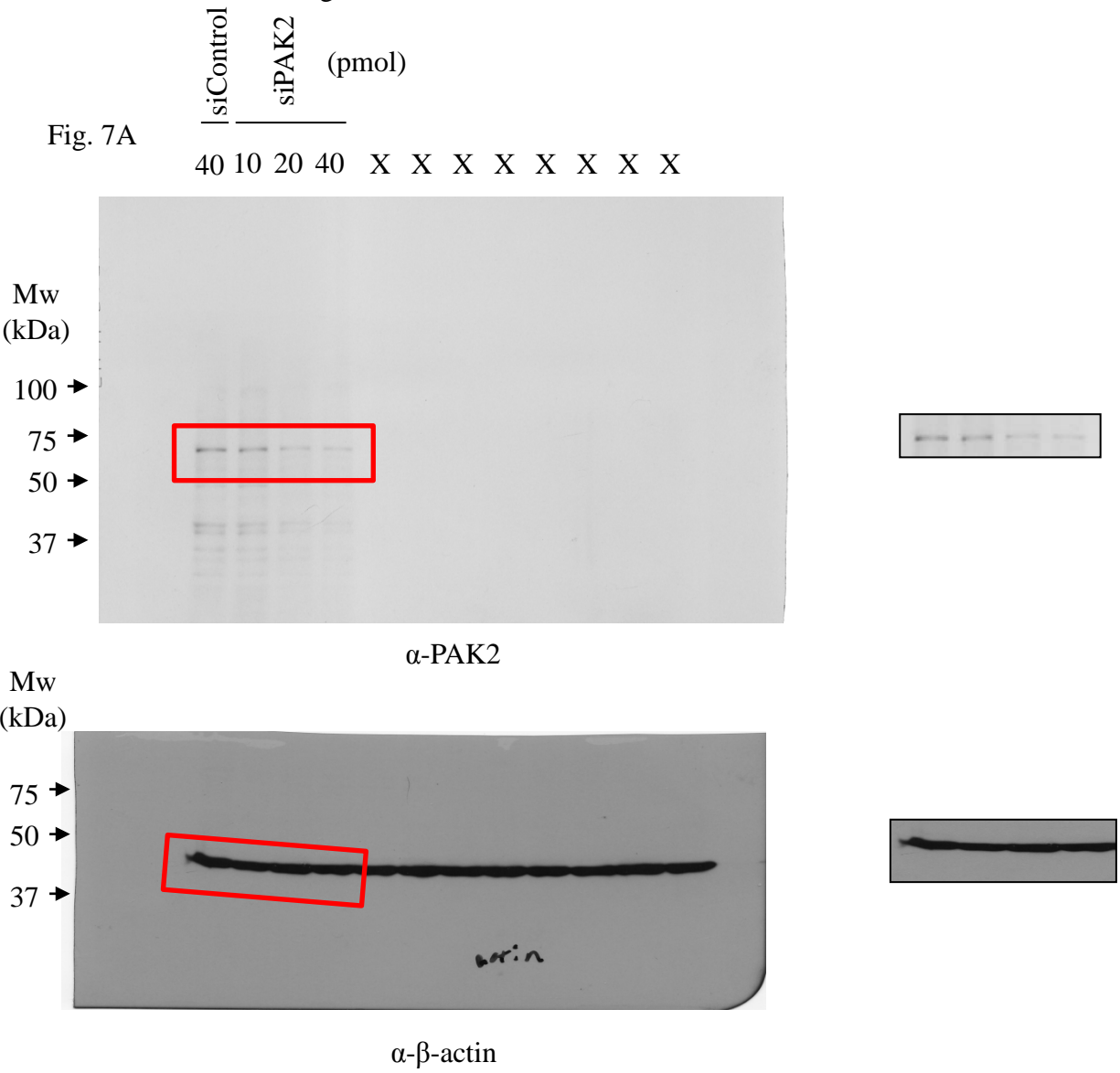

Supplement: S8 Fig — (PDF) [file pone.0246630.s008.pdf]

Fig. 7C

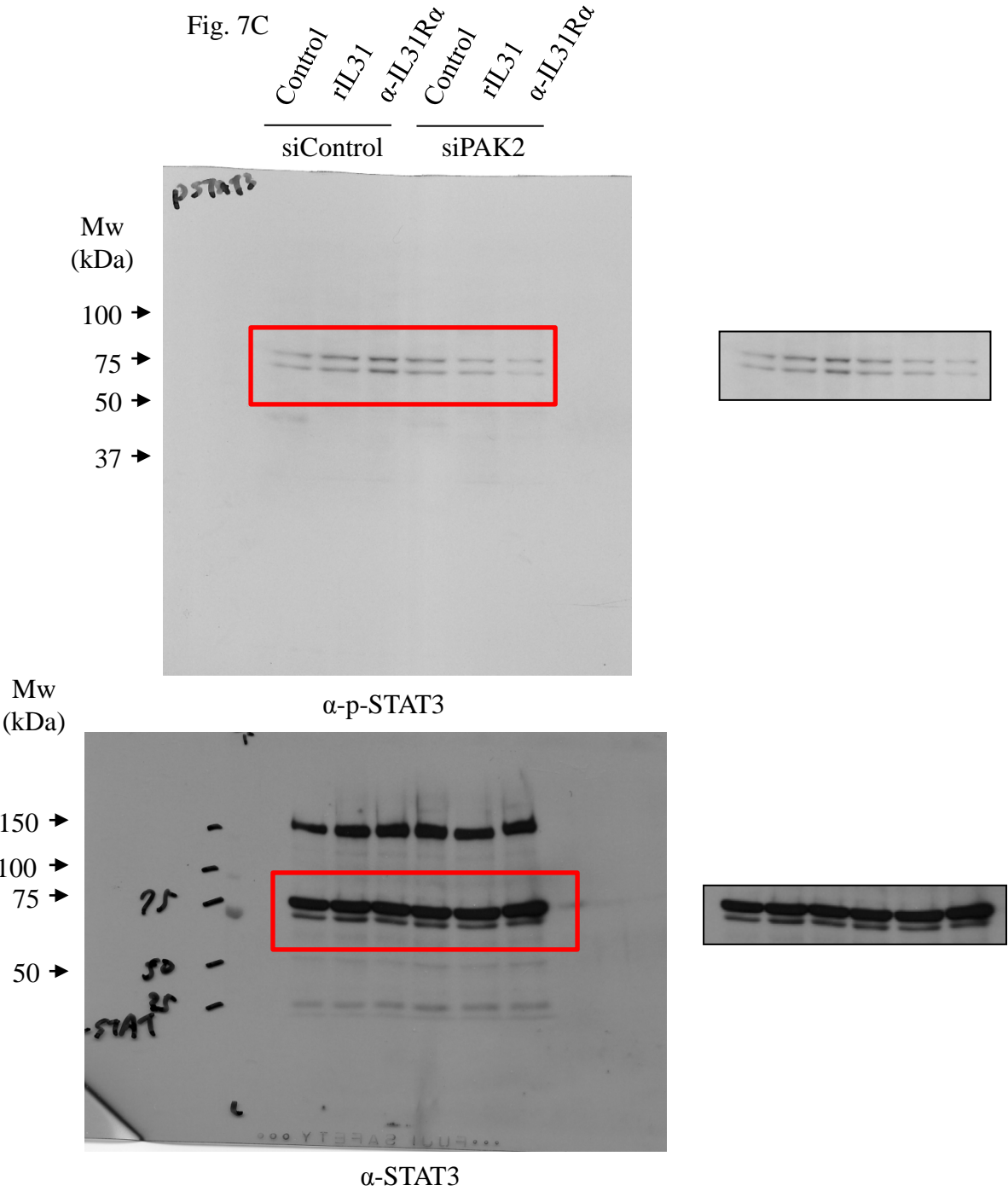

Supplement: S9 Fig — (PDF) [file pone.0246630.s009.pdf]

Fig. 8

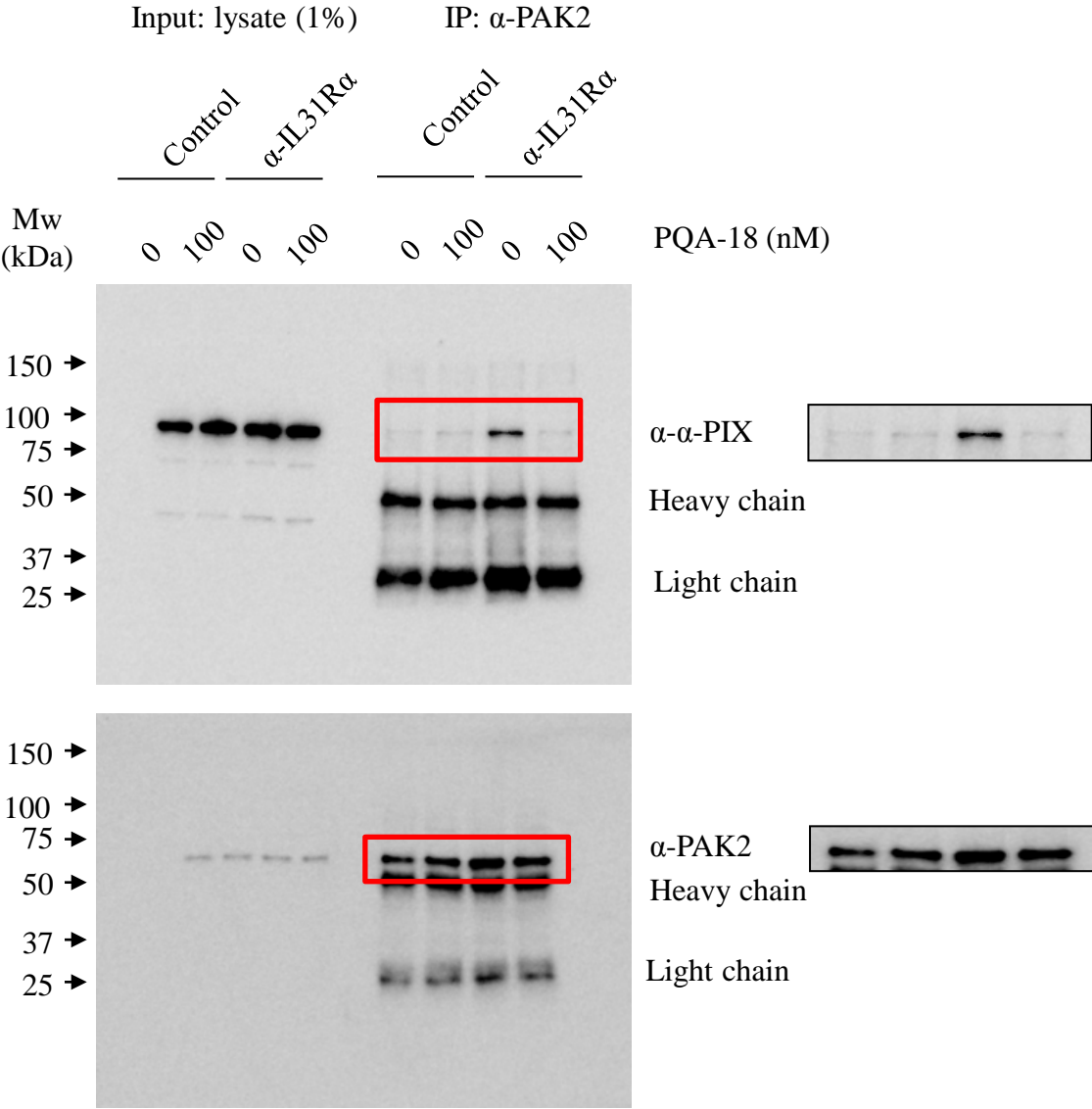

Supplement: S10 Fig — (PDF) [file pone.0246630.s010.pdf]
