# Supplementary material for: Prenylated quinolinecarboxylic acid compound-18 prevents sensory nerve fiber outgrowth through inhibition of the interleukin-31 pathway
Source: PLoS One. 2021 Feb 4;16(2):e0246630. doi: 10.1371/journal.pone.0246630 (PMC7861556; doi:10.1371/journal.pone.0246630)
Supplement: S1 Table — (PDF) [file pone.0246630.s011.pdf]

S1 Table minimal data set

Fig. 1A minimal data set

PGP9.5+ fiber density — Number of positive fibers/250000 μm2

	[1]	[2]	[3]	[4]	[5]	[6]	[7]	[8]	[9]	mice No.
(-)	40	39	63	47	60	45	60	63	22	
vehicle	39	27	40	26	37	54	55	44	13	
PQA-18	16	23	15	20	16	1	2	4	2	
FK506	24	41	39	39	44	27	25	50	13	

Fig 1B minimal data set

IL31Rα+ fiber density — Number of positive fibers/250000 μm2

	[1]	[2]	[3]	[4]	[5]	[6]	[7]	[8]	[9]	mice No.
(-)	43	82	104	61	42	90	21	125	33	
vehicle	114	42	34	41	93	52	20	38	28	
PQA-18	5	24	10	15	9	24	14	15	18	
FK506	57	27	25	55	26	44	31	15	45	

Fig 2 minimal data set

Nerve fiber length of IL31Rα+ cells (μm)

	1			2			3			Experiment No.
	1	2	3	4	5	6	7	8	9	well No.
Vehicle	316.8647	421.72751	217.81115	97.4	619	278	93.5	212.7	481	
rIL31	818.3843	530.2455	408.77525	464	407	452	431	1399	1042	
rIL31+ PQA-18	206.8437	364.2223	122.33096	249	33.2	69.9	251	24.96	277	
PQA-18	372.4475	584.49086	88.366871	38.6	302	336	233	103.7	260	

Fig 3C minimal data set

The ratio of pSTAT3 signal intensity / STAT3 signal intensity

rIL31 (ng/ml)	1	2	3	Experiment No.
0	0.27176	0.4881364	0.2268993	
0.1	0.259399	0.3646188	0.2464802	
1	0.438541	0.6080169	0.4337175	
10	0.712223	0.6019653	0.682657	
100	0.643877	0.6895177	0.6460978	

Fig 3D minimal data set

The ratio of pSTAT3 signal intensity / STAT3 signal intensity

α-IL31Rα (ng/ml	1	2	3	Experiment No.
0	0.446205	0.4677859	0.4333797	
10	0.520087	0.5510297	0.5236836	
50	0.864256	0.761479	0.7617817	

Fig 4 minimal data set

The ratio of pSTAT3 signal intensity / STAT3 signal intensity

	PQA-18 (nM)	1	2	3	Experiment No.
Control	0	0.472576	0.505547	0.4673388	
α-IL31Rα	0	0.62523	0.9556511	0.7140794	
α-IL31Rα	1	0.787672	0.6488525	0.6835925	
α-IL31Rα	10	0.579063	0.5266114	0.5863851	
α-IL31Rα	100	0.320732	0.4940064	0.3287427	
α-IL31Rα	1000	0.333676	0.5631653	0.3240861	

The ratio of pJAK2 signal intensity / JAK2 signal intensity

	PQA-18 (nM)	1	2	3	Experiment No.
Control	0	0.624608	0.6166735	0.6138751	
α-IL31Rα	0	0.871577	0.8574443	0.8445659	
α-IL31Rα	1	0.351358	0.3621349	0.3929143	
α-IL31Rα	10	0.405157	0.4124822	0.448723	
α-IL31Rα	100	0.499457	0.4867625	0.5535719	
α-IL31Rα	1000	0.445851	0.4435976	0.4426939	

The ratio of pPAK2 signal intensity / PAK2 signal intensity

	PQA-18 (nM)	1	2	3	Experiment No.
Control	0	0.334134	0.3720816	0.3136777	
α-IL31Rα	0	0.790032	0.8290717	0.7925981	
α-IL31Rα	1	0.416323	0.4175655	0.3739652	
α-IL31Rα	10	0.456063	0.46664	0.5356739	
α-IL31Rα	100	0.357612	0.3668209	0.509	
α-IL31Rα	1000	0.415063	0.4072811	0.5836007	

Fig 5 minimal data set Neurite outgrowth positive cells / total cells (%)

		1			2			3			Experiment No.
	PQA-18 (nM)	1	2	3	4	5	6	7	8	9	well No.
Control	0	0	7.6335878	7.751938	7.69	15.4	7.87	7.75	7.519	7.52	
α-IL31Rα	0	93.75	94.736842	52.631579	62.5	69.2	43.5	68.7	67.16	75.2	
α-IL31Rα	1	61.53846		38.461538	30.8	53.8	30.1	46.2	53.85	45.1	
α-IL31Rα	10	30.30303	23.076923	30.651341	23.1	30.7	53.8	38.5	30.65	37.6	
α-IL31Rα	100	23.05919	30.721966	23.023791	15.4	7.66	7.63	15.4	23.08	15	
α-IL31Rα	1000	15.34919	15.313936	0	0	7.63	7.75	0	0	7.52	

Fig 6 minimal data set

The ratio of pPAK2 signal intensity / PAK2 signal intensity

	FRAX597 (nM)	1	2	3	Experiment No.
Control	0	0.704339	0.6843724	0.7296274	
α-IL31Rα	0	1.21623	1.2237691	1.3758203	
α-IL31Rα	1	1.032585	1.0141383	1.5839801	
α-IL31Rα	10	0.884756	0.863831	0.9249098	
α-IL31Rα	100	0.7514	0.7153207	0.7279834	
α-IL31Rα	1000	0.278523	0.2392061	0.2112395	

Neurite outgrowth positive cells / total cells (%)

		1			2			3						Experiment No.		
	FRAX597 (nM)	1	2	3	4	5	6	7	8	9	10	11	12	13	14	well No.
Control	0	15.78947	0	10.526316	0	10.5	0	10.4	15.61	5.26						
α-IL31Rα	0	73.68421	52.273915	67.218201	52.6	78.9	94.7	84.2	42.11	100	89.5	36.8	100	68.4		
α-IL31Rα	1	21.05263	36.842105	63.157895	100	100	63.2	26.3	94.74	89.5	100	100	63	84.2	53	
α-IL31Rα	10	26.02811	57.894737	42.105263	31.6	26.2	36.8	37	68.42	31.6	63.2	36.8	21	26.3	27	
α-IL31Rα	100	5.263158	10.582011	31.578947	10.5	10.5	21.1	22	20.62	15.8	36.8	26.3	37	26.2		
α-IL31Rα	1000	5.263158	5.2631579	10.526316	10.5	10.5	10.5	10.5	10.53	5.26						

Fig 7B minimal data set Neurite outgrowth positive cells / total cells (%)

		1			2			3			Experiment No.
		1	2	3	4	5	6	7	8	9	well No.
siControl	Control	0	7.6923077	7.6335878	7.52	15.4	7.58	7.41	7.246	7.27	
siControl	rIL31 (ng/ml)	93.75	93.333333	50	45	68.7	30.7	68.2	61.54	67.7	
siControl	α-IL31Rα	94.33962	92.307692	38.461538	38.3	69.2	30.8	68.7	62.02	69.8	
siPAK2	Control	15.29052	15.337423	15.625	0	7.57	7.76	15.4	0	7.69	
siPAK2	rIL31 (ng/ml)	15.92357	15.25553	7.6923077	0	7.25	8.06	0	0	7.3	
siPAK2	α-IL31Rα	22.72727	31.007752	22.304833	15.5	7.56	7.63	15.2	7.874	14.7	

Fig 7C minimal data set

The ratio of pSTAT3 signal intensity / STAT3 signal intensity

		1	2	3	Experiment No.
siControl	Control	0.464721	0.5522186	0.4560587	
siControl	rIL31 (ng/ml)	0.726121	0.6892413	0.7154827	
siControl	α-IL31Rα	0.972666	0.7512375	0.967629	
siPAK2	Control	0.680257	0.479715	0.6656749	
siPAK2	rIL31 (ng/ml)	0.327833	0.2300948	0.3216619	
siPAK2	α-IL31Rα	0.167254	0.1076428	0.1623006	

Fig 8A minimal data set

The peak areas for PAK2

	PQA-18 (nM)	1	2	3	Experiment No.
Control	0	2873824	4515631	4313071	
Control	100	3752514	2940702	4017743	
α-IL31Rα	0	3620866	3851820	3997104	
α-IL31Rα	100	3438307	3493692	4758592	

The peak areas for GIT1

	PQA-18 (nM)	1	2	3	Experiment No.
Control	0	791639	862265	827945	
Control	100	777011	755591	869119	
α-IL31Rα	0	761259	720178	676783	
α-IL31Rα	100	700060	942667	781161	

The peak areas for GIT2

	PQA-18 (nM)	1	2	3	Experiment No.
Control	0	<10	<10	<10	
Control	100	<10	<10	<10	
α-IL31Rα	0	375512	<10	347547	
α-IL31Rα	100	<10	<10	<10	

The peak areas for α-PIX

	PQA-18 (nM)	1	2	3	Experiment No.
Control	0	<10	<10	<10	
Control	100	<10	<10	<10	
α-IL31Rα	0	409859	369539	412926	
α-IL31Rα	100	<10	308532	<10	

The peak areas for β-PIX

	PQA-18 (nM)	1	2	3	Experiment No.
Control	0	218933	315136	277993	
Control	100	307119	282350	302147	
α-IL31Rα	0	420654	453213	420202	
α-IL31Rα	100	547643	354807	<10	

Fig 8B

The ratio of α-PIX signal intensity / PAK2 signal intensity

	PQA-18 (nM)	1	2	3	Experiment No.
Control	0	0.21202	0.1748442	0.1816775	
Control	100	0.204829	0.2143059	0.2143868	
α-IL31Rα	0	0.907692	0.9713271	1.0415165	
α-IL31Rα	100	0.14384	0.1858421	0.1924074	
