# Supplementary material for: Prenylated quinolinecarboxylic acid compound-18 prevents sensory nerve fiber outgrowth through inhibition of the interleukin-31 pathway
Source: PLoS One. 2021 Feb 4;16(2):e0246630. doi: 10.1371/journal.pone.0246630 (PMC7861556; doi:10.1371/journal.pone.0246630)
Supplement: Attachment — Submitted filename: Response to Reviewers.docx [file pone.0246630.s012.docx]

Responses to editor’s comments:

*1. Please ensure that your manuscript meets PLOS ONE's style requirements, including those for file naming. The PLOS ONE style templates can be found at* [*https://journals.plos.org/plosone/s/file?id=wjVg/PLOSOne_formatting_sample_main_body.pdf*](https://journals.plos.org/plosone/s/file?id=wjVg/PLOSOne_formatting_sample_main_body.pdf) *and* [*https://journals.plos.org/plosone/s/file?id=ba62/PLOSOne_formatting_sample_title_authors_affiliations.pdf*](https://journals.plos.org/plosone/s/file?id=ba62/PLOSOne_formatting_sample_title_authors_affiliations.pdf)

Thank you for your comments. As suggested, text and file naming have been revised.

*2. In your Methods section, please provide additional information on the animal research and ensure you have included details on : (1) methods of sacrifice (2) methods of anesthesia and/or analgesia, and (2) efforts to alleviate suffering.*

Thank you for your comments. As suggested, the ‘methods’ section has been revised. In order to alleviate suffering, we used skilled cervical dislocation method. On the other hands, we did not use anesthesia to avoid its biological effects.

*3. In your Data Availability statement, you have not specified where the minimal data set underlying the results described in your manuscript can be found. PLOS defines a study's minimal data set as the underlying data used to reach the conclusions drawn in the manuscript and any additional data required to replicate the reported study findings in their entirety. All PLOS journals require that the minimal data set be made fully available. For more information about our data policy, please see* [*http://journals.plos.org/plosone/s/data-availability*](http://journals.plos.org/plosone/s/data-availability)*.*

*Upon re-submitting your revised manuscript, please upload your study’s minimal underlying data set as either Supporting Information files or to a stable, public repository and include the relevant URLs, DOIs, or accession numbers within your revised cover letter. For a list of acceptable repositories, please see* [*http://journals.plos.org/plosone/s/data-availability#loc-recommended-repositories*](http://journals.plos.org/plosone/s/data-availability#loc-recommended-repositories)*. Any potentially identifying patient information must be fully anonymized.*

*Important: If there are ethical or legal restrictions to sharing your data publicly, please explain these restrictions in detail. Please see our guidelines for more information on what we consider unacceptable restrictions to publicly sharing data:* [*http://journals.plos.org/plosone/s/data-availability#loc-unacceptable-data-access-restrictions*](http://journals.plos.org/plosone/s/data-availability#loc-unacceptable-data-access-restrictions)*. Note that it is not acceptable for the authors to be the sole named individuals responsible for ensuring data access.*

*We will update your Data Availability statement to reflect the information you provide in your cover letter.*

Thank you for your comments. As suggested, the Supporting Information has been revised. We have added our study’s minimal underlying data set to Supporting Information file.

*4. PLOS ONE now requires that authors provide the original uncropped and unadjusted images underlying all blot or gel results reported in a submission’s figures or Supporting Information files. This policy and the journal’s other requirements for blot/gel reporting and figure preparation are described in detail at* [*https://journals.plos.org/plosone/s/figures#loc-blot-and-gel-reporting-requirements*](https://journals.plos.org/plosone/s/figures#loc-blot-and-gel-reporting-requirements) *and* [*https://journals.plos.org/plosone/s/figures#loc-preparing-figures-from-image-files*](https://journals.plos.org/plosone/s/figures#loc-preparing-figures-from-image-files)*. When you submit your revised manuscript, please ensure that your figures adhere fully to these guidelines and provide the original underlying images for all blot or gel data reported in your submission. See the following link for instructions on providing the original image data:* [*https://journals.plos.org/plosone/s/figures#loc-original-images-for-blots-and-gels*](https://journals.plos.org/plosone/s/figures#loc-original-images-for-blots-and-gels)*.*

*In your cover letter, please note whether your blot/gel image data are in Supporting Information or posted at a public data repository, provide the repository URL if relevant, and provide specific details as to which raw blot/gel images, if any, are not available. Email us at* [*plosone@plos.org*](mailto:plosone@plos.org) *if you have any questions.*

Thank you for your comments. As suggested, the Supporting Information has been revised. We have added our all original full blot data set to Supporting Information file.

*5. We note that you have included the phrase “data not shown” in your manuscript. Unfortunately, this does not meet our data sharing requirements. PLOS does not permit references to inaccessible data. We require that authors provide all relevant data within the paper, Supporting Information files, or in an acceptable, public repository. Please add a citation to support this phrase or upload the data that corresponds with these findings to a stable repository (such as Figshare or Dryad) and provide and URLs, DOIs, or accession numbers that may be used to access these data. Or, if the data are not a core part of the research being presented in your study, we ask that you remove the phrase that refers to these data.*

Thank you for your comments. As suggested, the ‘discussion’ section has been revised. We have removed the sentence (page 21, lane 411).

*6. PLOS requires an ORCID iD for the corresponding author in Editorial Manager on papers submitted after December 6th, 2016. Please ensure that you have an ORCID iD and that it is validated in Editorial Manager. To do this, go to ‘Update my Information’ (in the upper left-hand corner of the main menu), and click on the Fetch/Validate link next to the ORCID field. This will take you to the ORCID site and allow you to create a new iD or authenticate a pre-existing iD in Editorial Manager. Please see the following video for instructions on linking an ORCID iD to your Editorial Manager account:* [*https://www.youtube.com/watch?v=_xcclfuvtxQ*](https://www.youtube.com/watch?v=_xcclfuvtxQ)

Thank you for your comments. As suggested, my Information has been revised.

*7. Please include captions for your Supporting Information files at the end of your manuscript, and update any in-text citations to match accordingly. Please see our Supporting Information guidelines for more information:* [*http://journals.plos.org/plosone/s/supporting-information*](about:blank)*.*

Thank you for your comments. As suggested, captions for Supporting Information files have been added to the end of the manuscript.

Responses to reviewer’s comments:

*Reviewer #1: Yoshimi Homma’s lab had previously reported that PQA-18, a derivative of Polysphondylium pseudo-candidum isolated small molecule Ppc-1, acts as an immunosuppressant and a PAK2 kinase inhibitor, could alleviate atopic dermatitis in mouse model (ref. 21). In the current study, the authors are trying to understand the mechanism of action of PQA-18 in relieving the skin defects in atopic dermatitis.
The authors chose to examine the IL-31/IL-31Rα pathway and provide data to show that PQA-18 decreased the cutaneous sensory nerve fiber density by inhibiting the expression of IL-31 receptor alpha in mouse skin. In cultured neuronal cells, PQA-18 inhibits IL-31 induced sensory nerve fiber outgrowth and neurite outgrowth by blocking IL-31Rα activation of the PAK2/JAK2/STAT3 pathway. Lastly, PQA-18 selectively blocks PAK2 interaction with GIT2 and α-PIX, which are thought to activate PAK2.*

*That PQA-18 acts as a potent immunosuppressant and effectively improves the skin pruritus condition in mouse AD model are potentially important findings. Experimental data for that PQA-18 inhibits IL-31/IL-31Rα pathway and prevents cutaneous nerve fibre growing is convincing. But the molecular mechanism described for PQA-18 inhibiting PAK2 kinase is less convincing. There are a several issues which the authors need to address.*

*Major points.*

*1. The authors believed that PAK2 is an important target of PQA-18 and based on MS data in Fig.7 they suggest that PQA-18 inhibited PAK2 activation by preventing its interaction with GIT2/α-PIX. However more direct evidence is required to demonstrate PQA-18 blocks PAK2 interaction with α-PIX (GIT2 is likely indirect) using a direct binding assay.*

Thank you for your comments. To incorporate this, we performed additional experiments using immunoprecipitation assay, and added findings to the ‘Results’ section (page 18, lane 374-377). Please refer new Fig 8B. We have changed previous labeled Fig 7 to new Fig 8.

*2. Since both PAK1 and PAK2 are expressed in neuronal cells (PAK2 being ubiquitous) the authors should clarify if PQA-18 has similar effects on PAK1 kinase.*

Thank you for your comments. As suggested, it is known that Neuro2A cells express both PAK1 and PAK2 (Ref 36). Although anti-phospho-PAK2 antibody recognizes both pSer141-PAK2 (61 kDa) and pSer144-PAK1 (68 kDa), we did not detect pSer144-PAK1 signal in our experimental condition (Figs 4 and 6). The pSer141-PAK2 signal was confirmed using anti-PAK2 antibody. Thus, it is conceivable that PAK2 rather than PAK1 is involved in the mechanism underlying the effect of PQA-18 on neurite outgrowth. Furthermore, we used Autodock Vina software (Le *et al*., *Scientific reports*, 2015) to analyze the PAK1 structure, and explore a binding pocket for PQA-18. However, we did not find suitable pocket. We will continue to analyze more details of the PAK-PQA interaction by considering dynamic structural changes, and report results on a separate paper.

*3. In Fig.2 (compare IL-31Rα images of vehicle vs PQA-18) the data does not support the claim that PQA-18 inhibits IL-31Ra expression in cutaneous nerve fibers. Cf. as stated on page 2 “PQA-18 alleviates cutaneous nerve fiber density and the expression of IL-31 receptor α (IL-31Rα) in the skin of Nc/Nga mice.” and page 11 “These results suggest that PQA-18 ointment, but not FK506 ointment, alleviates excessive cutaneous nerve fibre density and expression of IL-31Rα in the skin of Nc/Nga mice”.*

Thank you for your comments. As suggested, we have revised these sentences and removed the description of IL31Rα expression (page 2, lane 17; page 11, lane 217 and lane 222; page 22, lane 419).

*4. Do the authors think that PAK2 controls IL-31Rα gene expression in mouse skin?*

Thank you for your comments. As shown in Fig 2, IL31Rα was not only expressed in neuronal body but also nerve fiber structure in IL31-treated DRG neurons. Given the finding that PAK2 inhibitor PQA-18 suppresses IL31-induced nerve fiber development, we speculate that PAK2 may affect the expression of IL31Rα protein in sensory nerve fibers of lesional-skin. To confirm direct effects of PAK2 on IL31Rα gene expression *in vivo*, we are trying to produce conditional KO mice lacking PAK2, and would like to report their findings in the near future.

*Minor points:*

*1) Images of “lesional-” panel in Fig.1A and B were adjusted brighter than other panels. All images should be taken under the same exposure condition. Figure 1 legend does not introduce what are blue channel and green channel representing.*

Thank you for your comments. To incorporate this, Figs 1A, 1B and legend of Fig 1 have been revised (page 12, lane 228).

*2) Figure 1A and B, showed FK506 caused more than 40% reduction of PGP9.5+ and IL-31Rα fiber density, why do the authors think this is not significant? (see page 11, lines 219-220 and 225-226)*

Thank you for your comments. As a result of the one-way analysis of variance with Turkey-Kramer post-hoc comparisons, treatment with FK506 did not significantly reduce PGP9.5-positive and IL31Rα-positive fiber density as compared with none and vehicle-treatment mice. In addition, vaseline, the base of the vehicle ointment and FK506 ointment, has been reported to slightly relieve atopic dermatitis symptoms through induction of protective factors including filaggrin (Nomura and Kabashima, *J Allergy Clin Immunol,*2016). In fact, previous (Ref. 21) and the results from this study also show that the vehicle group tends to improve atopic symptoms slightly, if not significantly. Thus, we consider it important to compare with the vehicle group in *in vivo* analysis.

*3) Figure 1 legend: *p<0.01 should be *p<0.05*

As suggested, the legend of Fig 1 has been revised (page 12, lane 231).

*4) On page 18, this sentence is not clear. “PQA-18 significantly suppressed the development, while PQA-18 alone did not affect sensory nerve development.”*

As suggested, this sentence has been revised (page 12, lane 240-241).

*5) The authors need to clarify why DRG nerve fiber length in PQA-18 +rIL-31 treated DRG cells are shorter than PQA-18 alone and vehicle control in Fig.2 (see chart at bottom).*

Thank you for your comments. As suggested, the length of nerve fiber in DRG cells treated with PQA-18 +rIL-31 was not significant, but it was shorter than that of the vehicle control and PQA-18 alone group. It is possible that negative feedback loop of JAK/STAT signaling is involved in its mechanism. The systems include induction of suppressor of cytokine signal (SOCS) and activation of tyrosine phosphatases (Jiang *et al*., *Front Immunol*. 2017). Thus, we speculate that both the PQA-18 and the negative feedback loop induced by IL31 may be shortened because it suppresses neurite outgrowth in cooperation. We will examine IL31 signaling including negative feedback loop and report the findings.

*6) The authors need to explain why in Figure 4 PQA-18 inhibition of PAK2 and JAK2 phosphorylation is not dose dependent (over the conc used) however in Figure 5 PQA-18 inhibition of the neurite outgrowth is dose-dependent.*

Thank you for your comments. As suggested, we have discussed the differences in the effects of PQA-18 and FRAX597 on PAK2 inhibition mechanisms and neurite outgrowth, and added the content to the ‘discussion’ section (page 24, lane 465-477).

*7) On page 9, line 169, addition of 40 pmol siRNA is in what volume of medium? It is maybe better to express as concentration (similar issue in Fig.6).*

As suggested, the ‘methods’ section and new Fig 7A have been revised (page 9, lane 163). We have changed previous labeled Fig 6 to new Fig 7.

*Reviewer #2:*

*Ogura et al., (2016) showed that the prenylated quinolinecarboxylic acid derivative PQA-18 suppresses immune response, likely through inhibition of PAK2. This was based on studies showing that there were changes in cofilin phosphorylation upon PQA-18 treatment (which could be via effects of PAK2 on LIMK1). However the data presented then was not equivocal that PQA-18 inhibits PAK2 directly, although other studies in KO mice to point to PAK1 and PAK2 regulating the immune system.*

*In this new study it is noted that PQA-18 improves the skin pruritus condition in a mouse model. In order to demonstrate that PQA-18 works by inhibiting PAK2 (and likely PAK1) some additional experiment is needed. In the neuroblastoma cell line etc.. it would be important to validate their 'PAK2 inhibitor' a well characterized ATP mimetic of which FRAX are the best characterized. It should be noted that IPA-3 does not work in vivo and should not be used.*

*The effects of PQA-18 on the IL-31/ IL-31Rα pathway are quite convincing, and maybe clinically important. I highlight below some issues that need to be considered.*

*(1) The treatment with PAK2 SiRNA shown in Figure 6 leads to profound changes in neurite outgrowth while the PAK2 KD by western is not that convincing (A). To resolve whether the modest changes in PAK2 levels the authors need to use Frax 597/1036, which will strongly suppress the p-PAK Ser141 signal (which can be compared to PQA-18).*

Thank you for your comments. To incorporate this, we performed additional experiments using FRAX597, and added the findings to the ‘Results’ section (page 16, lane 317-321). Please refer new Figs 6A, 6B and 6C.

*(2) In Fig 3C the authors show Stat3 western blots. However since with no IL31 (lane 1) there is no observed pStat3 signal (bands) they need to present a different panel (ie one of the other blots which has been used to obtain average but not shown).*

Thank you for your comments. As suggested, Fig 3C has been revised.

*(3) Table 1. Data on the PAK2 interacting proteins should be more complete, and the 'top' MS derived set should be listed according to either enrichment relative to control or intensity / sequence coverage.*

Thank you for your comments. As suggested, Table 1 has been revised. Total coverage score and the number of detected unique peptides were added to Table 1.

*(4) The MS data which indicates that GIT2 and αPIX are present in the PAK complex is interesting (Fig 7). Based on current models, if PQA-18 inhibits PAK1/PAK2 directly one would expected that this would stabilize the PIX complex. So this new data is interesting but should be supported with PAK2 IP & western data (for say aPIX).*

Thank you for your comments. To incorporate this, we performed additional experiments using immunoprecipitation assay, and added the findings to the ‘Results’ section (page 18, lane 374-377). Please refer new Fig 8B. We have changed previous labeled Fig 7 to new Fig 8.

*(5) In the raw data the p-JAK shows 2 strong bands which seem to be co-regulated. What is the presumed identify of the top band?*

Thank you for your comments. We used anti-phospho-JAK2 (Tyr1008) antibody (CST) in this study. It is reported that cross-reactivity of this antibody is not observed with other JAK family members by immunoblotting. In addition, the top band (approximately 160 kDa) is not reacted with JAK2 antibody. Bands of 140 kDa and 120 kDa were observed with the JAK2 antibody (Fig 4 and S5 Fig full blot data), suggesting that it is not JAK family proteins. To identify the top band signal, we used amino acid sequence of JAK2 (DKVYYKV) and BLAST search. As a result, we selected macrophage-stimulating protein receptor (MST1R, RTK8, RON; 160 kDa) as candidates of top band. This protein expressed in DRG neurons is tyrosine kinase and involved in sensory nerve development (Franklin et al., *Mol Cell Neurosci*. 2009). We will clarify top bands using immunoprecipitation assay and LC/MS/MS.

*(6) In Figure 4 the anti-PAK2 data shows strong band at ~ 60 kDa with no other background bands. By contrast the anti-PAK2 in Figure 6 shows several background bands - why is there such a large difference in the WB? Are different Abs used?*

Thank you for your comments. As suggested, several background bands for PAK2 were detected in new Fig 7. However, when we performed experiments using PQA-18 and FRAX597, these background bands were not detected (Fig 4, new Fig 6A, S6 Fig and S7 Fig). Because we performed experiments at same antibody, it is possible that non-specific signals were induced by siRNA or electroporation method. We will try to examine other transfection reagents such as Lipofectaime2000 (Invitrogen) and Fugene HD (Promega) to avoid non-specific signal.

*(7) The identity of the various antibodies used for analysis should be better defined in the figure legends (for example Fig 4) and ideally on the figures themselves. cf. The specifics of sites for phosphorylated sites in STAT3, JAK2, and PAK2.*

Thank you for your comments. As suggested, figure legends have been revised. Please refer the legends of Figs 3 and 4, new Figs 6 and 7 (page 14, lane 271-272; page 14, lane 289-290; page 16, lane 325; page 17, lane 351).
