# Supplementary material for: Prenylated quinolinecarboxylic acid compound-18 prevents sensory nerve fiber outgrowth through inhibition of the interleukin-31 pathway
Source: PLoS One. 2021 Feb 4;16(2):e0246630. doi: 10.1371/journal.pone.0246630 (PMC7861556; doi:10.1371/journal.pone.0246630)
Supplement: Attachment — Submitted filename: Response to Reviewers.docx [file pone.0246630.s013.docx]

Responses to editor’s comments:

*1. There is one key issue outstanding regarding the new Figure 6 that will require the authors expanding/rewriting that section, namely that they have used Frax597 at a sub-optimal concentration. The reason for doing this might be to avoid 'off-target' effects of Frax597 which are documented (to avoid affecting other kinases).*

*In Figure 6 I note you observe ~ 50% inhibition of PAK2 pS141 in their cells using 1 uM Frax597 with more substantive effect in cell assay. Thus the author should have considered 2 and 5 uM Frax597 doses. Please revise the MS to reflect the reasons why the 1 uM maximum (50% inhibition) was chosen for the neurite outgrowth assay (and p-PAK2 tested I think after for 30 min, rather than longer times, cf. 24/48h).*

*Admittedly the literature is a mess with respect to the proper concentration and timing to block PAK activity in cells. While the in vitro Ki is ~ 10-50 nM the effective dosing of cells with Frax579 usually requires 1-5 uM (ie 100 times more).*

*Licciulli et al., (2013) which the authors quote indeed indicates that pS141/4 signal was suppressed the cellular inhibition in SC4 cells in the range 0.5 -1 uM after 2h treatment. However this may reflect sensitivity of SC4 or a purer source of Frax579. In a 2015 paper (Oncotarget. Jul 10;6(19):16981-97) the authors use a 2h treatment of 2 uM Frax597 to effectively block both pS144 /141 signals.*

*Please submit a revised version of the manuscript that addresses the this point.*

Thank you for your comments. To incorporate this, we performed additional experiments using FRAX597 at 1, 2, and 5 μM, and added the findings of FRAX597 at 1 μM to the Figs 6A, 6B and 6C. The Supporting Information has been also revised.

As shown in Fig. R1, we observed that treatment with FRAX597 at 1, 2, and 5 μM (high concentration) for 30 min remarkably reduced phosphorylation of PAK2 in Neuro2A cells. However, we also observed that treatment with Frax597 at 2 and 5 μM for 48 h significantly reduced cell viability in Neuro2A cells (Fig. R2, Shakespear, Ogura *et al*., *Neurochem Res*., 2020). Thus, we did not add the findings of FRAX597 at 2 and 5 μM to the Fig 6. It has been reported that FRAX597 at high concentration can inhibit other kinases such as YES1 and RET (26). Given the reports that YES1 (Src family tyrosine kinase; Shani V *et al*., *J Mol Neurosci*., 2009) and RET (neurotrophin receptor tyrosine kinase; Tansey MG *et al*., *Neuron*, 2000) play an important role in neuronal survival, it is conceivable that FRAX597 at 2 and 5 μM may affect cell survival through inhibition of their tyrosine kinases in Neuro2A cells.

Responses to reviewer’s comments:

*Reviewer #1:Authors have addressed most of the concerns I raised earlier. In my opinion, provided the data on PAK inhibition in Figure 6 is better explained, the manuscript after minor revisions is therefore publishable.*

Thank you for your comments. As suggested, we performed additional experiments using FRAX597 at 1, 2, and 5 μM (Figs R1 and R2), and added the findings of FRAX597 at 1 μM to the Figs 6A, 6B and 6C. Since treatment with FRAX597 at 2 and 5 μM significantly reduced cell viability in Neuro2A cells, we did not add the results to the Fig 6.
